# Cognitive and Affective Processes Associated with Social Biases

**DOI:** 10.1093/ijnp/pyab022

**Published:** 2021-04-30

**Authors:** Asuka Kaneko, Yui Asaoka, Young-A Lee, Yukiori Goto

**Affiliations:** 1 Primate Research Institute, Kyoto University, Inuyama, Aichi, Japan; 2 Department of Food Science and Nutrition, Daegu Catholic University, Gyeongsan,South Korea

**Keywords:** Cognitive bias, negative affects, theory of mind, dual process theory, autism spectrum disorder

## Abstract

**Background:**

Our social activities are quite often erroneous and irrational, based on biased judgements and decision-making, known as social biases. However, the cognitive and affective processes that produce such biases remain largely unknown. In this study, we investigated associations between social schemas, such as social judgment and conformity, entailing social biases and psychological measurements relevant to cognitive and affective functions.

**Method:**

This study recruited 42 healthy adult subjects. A psychological test and a questionnaire were administered to assess biased social judgements by superficial attributes and social conformity by adherence to social norms, respectively, along with additional questionnaires and psychological tests for cognitive and affective measurements, including negative affects, autistic traits, and Theory of Mind (ToM). Associations of social judgment and conformity with cognitive and affective functions were examined using a multiple regression analysis and structural equation modeling.

**Results:**

Anxiety and the cognitive realm of ToM were mutually associated with both social judgments and conformity, although social judgements and conformity were still independent processes. Social judgements were also associated with autistic traits and the affective realm of ToM, whereas social conformity was associated with negative affects other than anxiety and an intuitive decision-making style.

**Conclusions:**

These results suggest that ToM and negative affects may play important roles in social judgements and conformity, and the social biases connoted in these social schemas.

**Significance Statement**

Our social activities are quite often erroneous and irrational, based on biased judgements and decision-making, known as social biases. Some representative social biases are the halo effect, articulated by Thorndike 100 years ago, which is making biased judgments of others based on their superficial traits, and social conformity, articulated by Asch in the 1950s, which is a tendency to fit one’s beliefs and behaviors to social groups or society itself (as social norms). Our study has demonstrated that negative affects, such as anxiety, and Theory of Mind are associated with social schemas, such as social judgments and conformity, entailing social biases. Social judgments and conformity also appear to be rather selectively associated with cognitive and affective processes, respectively, suggesting that social biases may be engendered by multiple independent processes. These findings are important in showing for the first time the cognitive and affective mechanisms that are involved in social biases.

## Introduction

Information processing in the human brain is thought to roughly be divided into intuitive versus analytical processes, known as the dual process theory (Evans, 2008; Kahneman, 2011; Grayot, 2020). The intuitive process is fast and autonomous and is primarily associated with affective functions, such as recognition of the emotional facial expressions of others (Evans, 2008; Kahneman, 2011; Grayot, 2020). In contrast, the analytical process is slow and effortful and is associated with cognitive functions that inhibit the intuitive process (Evans, 2008; Kahneman, 2011; Grayot, 2020). The intuitive process, although important for quick and adaptive decision-making, yields systematic but allegedly erroneous patterns of responses in judgments and decision-making, known as cognitive biases (Frederick, 2005; Kahneman, 2011; Toplak et al., 2011). Studies have demonstrated that negative affects, such as anxiety and stress, shift the balance of processes toward more intuitive responses, facilitating biased judgements and decision-making (Bodenhausen et al., 1994; Harding et al., 2004; Engelmann and Hare, 2018), which appears to be consistent with evolutionary views (Shah and Oppenheimer, 2008; Gigerenzer and Gaissmaier, 2011; Santos and Rosati, 2015); for instance, life-threatening environments where stress and anxiety are heightened require more hasty and impulsive fight-or-flight decision-making.

Cognitive biases could engender considerable influence on our social activities, compelling our socially relevant judgements and decision-making to quite often be irrational. Some representative examples of such social biases are the halo effect and social conformity. The halo effect, articulated by Thorndike 100 years ago (Thorndike, 1920), is autonomous social judgements of others based on their superficial attributes (appearance). Social conformity refers to the tendency of changing attitudes, beliefs, and behaviors to align with those of small groups of people or society as a whole: that is, social norms (Asch, 1951). Although these social biases have been noticed for a quite long time now, the cognitive (analytical) and affective (intuitive) mechanisms that produce such social biases have remained unclear.

Autism spectrum disorder (ASD) is a neurodevelopmental disorder with social communication deficits (Lord and Bishop, 2015). In the view of the dual process theory, ASD appears to be characterized by impaired intuitive but facilitated analytical processes. Thus, studies have shown that subjects with ASD exhibit impairments in recognition of the emotional facial expressions of others (Wang et al., 2007; Uljarevic and Hamilton, 2013; Ogawa et al., 2017), which depend on the intuitive process, whereas they are better at mathematical skills (Iuculano et al., 2014), which depend on the analytical process. These findings are consistent with those of a study directly investigating cognitive biases in ASD subjects using the cognitive reflection test, which reported less-biased responses in ASD subjects than healthy subjects (Brosnan et al., 2016). Deficits of the Theory of Mind (ToM) have also been implicated to underlie ASD (Baron-Cohen et al., 1985; Frith, 2012). Accumulating evidence suggests that the ToM could be divided into the cognitive and affective realms, representing abilities to attribute thoughts and beliefs of others and emotional states of others, respectively (Shamay-Tsoory and Aharon-Peretz, 2007; Bodden et al., 2010; Terrien et al., 2014; Adjeroud et al., 2016). How social biases in social judgments and conformity may be altered in people with ASD and associated with the cognitive and affective ToM have largely been unknown.

Collectively, in this exploratory study, we investigated associations between social schemas entailing social biases—that is, social judgments and conformity—and several cognitive and affective measurements that might be involved in the schemas, such as negative affects, autistic traits, decision-making styles, and ToM, in healthy adult subjects using a multiple regression analysis and structural equation modeling, to elucidate some insights about the cognitive and affective mechanisms that produce social biases.

## METHODS

### Subjects

This study was conducted in accordance with the Declaration of Helsinki and the Ethical Guidelines for Medical and Health Research Involving Human Subjects by the Japanese Ministry of Health, Labour, and Welfare. All experimental procedures were approved by the Human Research Ethics Committee of Kyoto University Primate Research Institute and the Ethics Committee of Kyowa Hospital. Upon enrolling into the study, written informed consent was obtained from all participants.

A total of 42 healthy adult subjects (20 males [23.6 ± 0.45 years old] and 22 females [25.0 ± 1.56 years old]), who were mostly undergraduate and graduate students at Kyoto University, were recruited by advertisements. No subjects were related to each other. The inclusion criteria for subjects were being raised in a Japanese cultural background and an age between 18 and 60 years. Subjects who had ever had a diagnosis of any neurological or psychiatric disorder; whose estimated full-scale intelligence quotient (eFIQ), which was assessed with a short form of the Japanese version of Wechsler Adult Intelligence Scale (WAIS)-III consisting of the Information test in the Verbal Comprehension Index and the Picture Completion test in the Perceptual Organization Index (Kobayashi et al., 1993; Dairoku et al., 2008) was below 60; or who were unable to give informed consent for any reason were excluded. No statistically significant difference in either age (23.6 ± 0.45 and 25.0 ± 1.56 years old for male and female subjects, respectively; t_40_ = −0.858; *P* = .396) or eFIQ (105.4 ± 2.53 and 105.6 ± 1.89 for male and female subjects, respectively; t_40_ = −0.061; *P* = .951) was observed between male and female subjects; therefore, no covariable was considered in any of the following statistical analyses. The sample structure regarding sex, age, and eFIQ is also summarized in Supplementary Table S1.

### Questionnaires

An assortment of questionnaires was utilized to evaluate (1) social norm conformity; (2) autistic traits; (3) negative affects; and (4) intuitive/analytical decision-making styles. These questionnaires were administered online through a website.

As a measure of social conformity, the Social Norm (SN) Questionnaire, which was developed by Rankin (Rankin, 2008; Kramer et al., 2014) for neurobehavioral evaluations in neurodegenerative disease patients, was administered. The SN Questionnaire consists of 22 items about social rules in the context of one’s own home country cultural background, in which the subject determines whether the behaviors described in the questionnaire are socially acceptable to perform. The questionnaire was translated into Japanese by the authors. Considering unfamiliarity of the subject matter for Japanese participants due to Western and Japanese cultural difference, 1 minor change was made in 1 question: “eat ribs with your fingers” was changed into “eat sushi with your fingers.” Eating sushi with your fingers is a traditionally acceptable table manner in Japan, although many modern Japanese people do not follow this practice any more. Participants answered yes or no for each question. A total score (maximum score = 22) indicated the tendency to adhere to social norms, with a higher score indicating stricter adherence, thereby thought to be stronger social conformity.

To examine what psychological aspects might be involved in social judgments and conformity, autistic traits; negative affects, such as stress, depression, and anxiety; and analytical versus intuitive decision-making styles were also evaluated with questionnaire surveys. The autistic traits of participants were evaluated with the Autism-Spectrum Quotient (AQ) using a Japanese translation (Baron-Cohen et al., 2001; Wakabayashi et al., 2006). The negative affects were assessed using the 21-item version of the Depression Anxiety Stress Scale (DASS-21; Lovibond and Lovibond, 1995). A total score on the DASS-21 (DASS_t_), which reflected overall negative affects, could be divided into the subscales for depression (DASS_d_), anxiety (DASS_a_), and stress (DASS_s_). The 40-item Rational-Experiential Inventory (REI) was also administered to assess decision-making styles, with the 2 main scales for rationality (REI_r_) and experientiality (REI_e_) further divided into 4 subscales by ability and engagement, where ability reflected how one can think intuitively or analytically and engagement indicated a tendency to think intuitively or analytically (Pacini and Epstein, 1999).

### Social Bias Test

**Figure 1.**
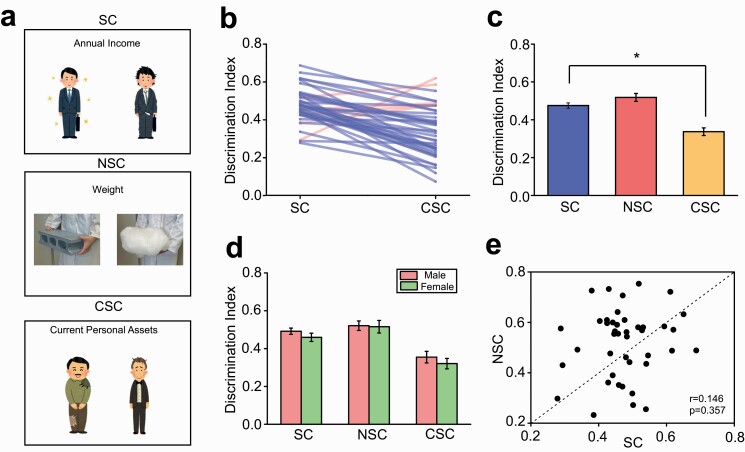
Social bias test. (A) Diagrams illustrating examples of the SC, NSC, and CSC, where the faces and attires are contradicted for judgments. (B) A graph illustrating the individual changes on the discrimination index between the SC and CSC. The blue and red lines indicate those exhibiting decreases on the discrimination index in the CSC compared to SC and vice versa, respectively. (C) A graph illustrating the average changes on the discrimination index in the SC, NSC, and CSC. *P < .001. (D) A graph illustrating the changes on the discrimination index separately for male and female subjects. (E) A scatter plot showing the relationship on the discrimination index between the SC and NSC. Abbreviations: CSC, conflicted social condition; NSC, nonsocial judgement condition; SC, social condition.

To examine social judgements on others by appearance, we conceived the Social Bias (SB) test, which is similar to the Social Rank Recognition test we had developed and used in our previous study (Ogawa et al., 2019). The SB test consists of a total of 12 images, which are comprised of 4 images each for the social condition (SC), nonsocial condition (NSC), and conflicted social condition (CSC). Examples of each condition are illustrated in Figure 1A. Each image described a pair of persons in the SC and CSC or a pair of objects in the NSC, with 1 positioned on the left and the other on the right side of the image. These persons or objects in the images of SC or NSC, respectively, were characterized by impressions of distinctions, whereas in the images of the CSC, the appearances conflicted with what would be expected for the persons in the images. In addition, each image contained a word or words (e.g., annual income, weight, etc.) on the top that instructed participants to rate for the persons or objects in the image using numerical numbers. The rating had no upper or lower limits, so that the rated numeric values could be anything that participants came up with. Social and nonsocial judgments on the pair of persons in the SC/CSC and the pair of objects in the NSC, respectively, were quantified as absolute differences of the values given between the pair. Since values were substantially different between each image, they was normalized, which we consequently expressed as the Discrimination Index (DI) = |(N_1_ − N_2_)|/(N_1_ + N_2_), where N_1_ and N_2_ were the numerical numbers that participants rated for a pair of persons or objects in each image, with a higher DI indicating thought to reflect more biases in judgements.

### Sucrose–Color Preference Test

In this study, we also conceived the Sucrose–Color Preference (SCP) test, which was similar to the tests created by Lavin and Lawless (1998) and Bayarri and colleagues (2001), to further evaluate nonsocial judgments by appearance (Supplementary Figure S1A). In this test, a 7.0% sucrose solution with 8 different colors (Pink [x: 0.374, y: 0.362, Y: 92.1] in the CIE xyY color space [Smith and Guild, 1932], Purple [x: 0.365, y: 0.341, Y: 70.5], Red [x: 0.529, y: 0.364, Y: 50.3], Yellow [x: 0.444, y: 0.493, Y: 120], Green [x: 0.310, y: 0.480, Y: 33.5], Blue [x: 0.254, y: 0.287, Y: 39.4], Brown [x: 0.450, y: 0.393, Y: 25.1], and Black [x: 0.352, y: 0.385, Y: 16.4]; solutions colored with organic food color dyes) were prepared. All 8 solution colors were presented to participants at once with a pseudo-random order of colors from the left to right. Participants were first asked to choose the 1 color that they liked the most. Then, participants were instructed to drink 2.0 mL of each of the solutions in any order that they chose, followed by answering which solution was the sweetest and which was the least sweet. Participants could also decide to answer that all solutions were equally sweet. Numerical quantifications of the colors in the CIE xyY color space were attained using the Luminance Color Meter CS-100A (Konica-Minolta, Tokyo, Japan). Color distances were measured between the color that a participant liked the most and the colors that the participant said were the sweetest (SCP_m_) and the least sweet (SCP_l_).

### Yoni Test

The Yoni test, which was originally developed by Shamay-Tsoory and Aharon-Peretz (2007) and has now been used in various studies (Bodden et al., 2010; Terrien et al., 2014; Adjeroud et al., 2016), assesses the ToM, an ability to infer the mental states of others. In the test, participants are instructed to answer what the character named “Yoni” was inferring based on his eye gaze, facial expression, or both. The test consists of 3 conditions. The cognitive ToM condition asks the participant to determine Yoni’s mental states based on emotionally neutral cues (eye gaze only), whereas the affective ToM condition requires mental inference of Yoni with emotional information (facial expression in addition to eye gaze). In addition, the physical ToM condition was conducted as a control, in which participants were asked to report physical attributes of the character (e.g., positions of Yoni on the screen). Each condition was further divided into first- and second-order inferences. The first-order inference required an analysis of the mental states or physical attributes of Yoni himself, whereas the second-order condition required understanding what Yoni was inferring about the beliefs or emotional states of another character or considering the physical attributes of Yoni in relation to another character.

**Figure 2.**
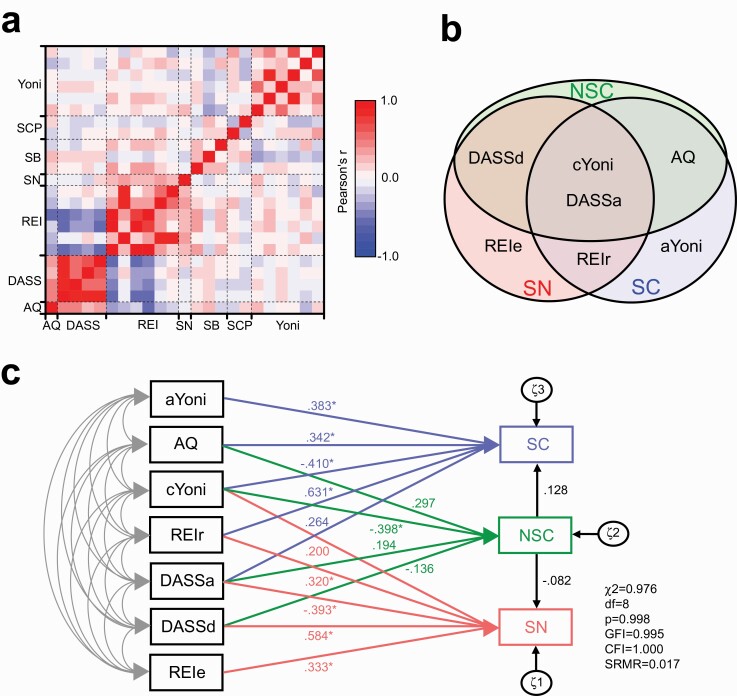
Path analysis for social and nonsocial judgments and social conformity. (A) A correlation matrix of all measurements examined in the study. Strengths of linear correlations (Pearson’s r) between the measurements are color-coded. (B) A Venn diagram illustrating the variables associated with SC, NSC, and SN Questionnaire in the multiple regression analysis. (C) A path diagram illustrating the nonlayered structure model with the best-fitting values of the chi-square statistic, GFI, CFI, and SRMR that have met with the criteria for a just-identified model. *P < .05 for path coefficients. Abbreviations: AQ, Autism-Spectrum Quotient; aYoni, affective assessment of the Theory of Mind; cYoni, cognitive assessment of the Theory of Mind; CFI, comparative fit index; DASS, Depression Anxiety Stress Scale; DASS_a_, Depression Anxiety Stress Scale anxiety subscale; DASS_d_, Depression Anxiety Stress Scale depression subscale; GFI, goodness-of-fit indices; NSC, nonsocial judgement condition; REI, Rational-Experiential Inventory; REI_e_, Rational-Experiential Inventory experientiality scale; REI_r_, Rational-Experiential Inventory rationality scale; SB, Social Bias test; SC, social condition; SCP, Sucrose–Color Preference; SN, Social Norm; SRMR, standardized root mean square residual.

The Yoni test was administered on an LCD computer monitor using Inquisit Lab (Millisecond Software, Seattle, WA). In this test, the face of Yoni, with his eye gaze toward 1 corner of the screen, with or without a facial expression, appeared in the center of the screen, along with 4 objects (animals, fruits, etc. for the first-order ToM) or other’s faces next to the objects (for the second-order ToM) in the corners of the screen, with 1 object at each corner (for examples, see Figure 2 of Shamay-Tsoory and Aharon-Peretz, 2007). Participants were instructed to state what Yoni was inferring by using the computer mouse to point to the corner of the screen as fast as they could. A verbal cue (e.g., Yoni is thinking of _____.) shown at the top of the screen in the test was translated into Japanese by the experimenters and presented to participants in this study.

A single session with a total of 98 trials, which were divided into 12 cognitive (eye gaze only), 12 affective (facial expression with eye gaze), and 8 physical (a position of Yoni’s face is at 1 corner in the screen) first-order ToM trials, as well as 24 cognitive, 36 affective, and 6 physical second-order ToM trials, were administered to each participant, in the following 3 phases. Phase A consisted of 8 trials each of the first-order cognitive, affective, and physical conditions in a pseudorandom order. Phase A was followed by Phase B, in which 4 trials each of the first-order cognitive and affective conditions and 24 trials of the second-order affective condition were given in a pseudorandom order. Then, Phase C consisted of 24, 12, and 8 trials of the second-order cognitive, affective, and physical conditions, respectively, in a pseudorandom order. There was no interval between trials and phases. Most participants completed a session within 10 minutes. Accuracy (the percentages of correct responses in the cognitive [cYoni], affective [aYoni], and physical [pYoni] conditions) and reaction time (cYoniRT, aYoniRT, pYoniRT) were measured.

### Data Analysis

Investigators who were not blinded to the experimental conditions collected data and conducted the statistical analysis. The statistical analysis was conducted using STATISTICA 7.1 (StatSoft, Inc., Tulsa, OK, USA). A (an asymptotic) probability value of *P* < .05 was considered to be statistically significant.

A statistical analysis of the data was conducted using both parametric and nonparametric tests, depending on normality (whether measurements were fit with normal distribution or not using the Shapiro-Wilk test; Supplementary Tables S2 and S3). An unpaired *t*-test or Mann-Whitney U test was used for statistical comparisons between male and female subjects in each measurement. In the SB test, a comparison of variables (SC, NSC, and CSC) was conducted using a 1-way analysis of variance (ANOVA) with repeated measures, followed by post hoc pair-wise comparisons using the Tukey test, whereas a comparison of variables (cognitive, affective, and physical) in the Yoni test was conducted using a Friedman ANOVA with post hoc pair-wise comparisons using the Wilcoxon rank sum test. The Wilcoxon rank sum test was also used to compare variables (SCP_m_ and SCP_l_) in the SCP test.

In the data set, several outliers (n = 1 in the SCP test and n = 3 in the Yoni test) were included, which were removed based on the following procedures, especially since outliers could cause serious problems in the multiple regression analysis subsequently conducted as described below. In the measurement with a normal distribution, outliers whose values were larger or smaller than 3 times the standard deviation from the mean (mean ± 3SD) were removed, whereas in the measurement with a nonnormal distribution, the median absolute deviation (MAD) method was used to remove outliers (Leys et al., 2013), so that the outliers whose values were larger or smaller than 3 times the median absolute deviation from the median (M ± 3MAD) were removed.

To examine correlations between measurements, linear correlations were conducted for all possible pairs of the measurements in a round-robin, and a correlation matrix was created with Pearson’s r (Figure 2A). A multiple regression analysis was then conducted with selected measurements. For the dependent variables, SC (social judgements), NSC (nonsocial judgments), and SN (conformity to social norms) were selected as factors entailing social biases. The independent variables included AQ, DASS_a,_ DASS_d,_ DASS_s_, cYoni, aYoni, REI_r_, REI_e_, SCP_m_, and SCP_l_. Multiple regression was conducted using the backward stepwise method to obtain the best regression models, along with the contribution criteria of the variables in the regression equations for F = 1.0 and 11.0 to determine how significant contributions of the variables in the regression equations had to be in order for them to be added to or removed from the equations. Coefficients of multiple correlations (R) and coefficients of multiple and adjusted determinations (R^2^ and adjR^2^), F-values, and resulting *P* values were considered for the goodness of fit for the models. A residual analysis was further conducted to evaluate adequacy of the models. For multicollinearity, tolerance and the variance inflation factor (VIF) were examined for each independent variable (O’Brien, 2007; Oke et al., 2019), with instances where the tolerance was smaller than 0.4 or the VIF was larger than 2.5 set for the criteria to not include the variables. One of the variables, DASS_t_, met with this criteria for the collinearity with DASS_a,_ DASS_d,_ and DASS_s_, and was dropped from the regression analysis. The Durbin-Watson statistic, whose value between 1.5 and 2.5 was thought to be relatively normal, was utilized to evaluate the problem of autocorrelation (Kramer, 1985). Multivariate normality was evaluated by a normal distribution of residuals with the Shapiro-Wilk test (Harrell and Lee, 1985). In addition, Cook’s distance for individual cases was examined to screen out the possibility of an outlier case (larger than 1.0) in the model (Oyeyemi et al., 2015). The assumption of homoscedasticity was also evaluated by Levene’s test on standardized residuals versus standardized predicted values in each model.

Path analysis with structural equation modeling was conducted to further evaluate how representative variables, such as AQ, DASS_a_, DASS_d_, REI_r_, REI_e_, cYoni, and aYoni, contributed to SC, NSC, and SN. Several *a priori* models (theories), such as models with layered (some independent variables were associated with other independent variables, which in turn were associated with the dependent variables) and nonlayered (all independent variables were directly associated with the dependent variables) structures, were considered. To evaluate how well such *a priori* models fit the data—that is, the model fit—several absolute fit indices were assessed. Included in these indices were the χ ^2^ statistic, goodness-of-fit indices (GFI), a comparative fit index (CFI), and the standardized root mean square residual (SRMR). The criteria for an acceptable, just-identified model that we utilized were a low χ ^2^ relative to degrees of freedom with an insignificant *P* value (*P* > .05) along with a GFI value greater than 0.95 and a CFI value greater than 0.96, with a SRMR of 0.09 or lower (i.e., Hu and Bentler’s 2-Index Presentation Strategy; Hu and Bentler, 1999; Hooper et al., 2008). Once the acceptable model was established, path coefficients between variables in the model were appraised.

### Data Availability

The data sets generated and analyzed during the current study are available from the corresponding author upon reasonable request.

## RESULTS

### Social Bias Test

In the SB test, participants consistently rated the differences (i.e., the DI) of the pairs of persons in SC or objects in NSC at approximately around 0.5, which was significantly decreased in the CSC (F_2,82_ = 30.6; *P* < .001; *P* < .001 for SC vs. CSC; Figure 1B and C), suggesting that participants made biased judgments in this test. Further analysis unveiled no sex difference in any condition (Figure 1D). There was also no correlation of the DI between the social and nonsocial conditions (Figure 1E), suggesting that social and nonsocial judgement processes may be independent from each other.

### SN Questionnaire

The score on the SN Questionnaire was 17.6 ± 0.21 in the current study group (Supplementary Table S2). This score was slightly lower than that in the construct validity study with a larger sample size that was conducted in the United States (Rankin, 2008; Kramer et al., 2014), but was still comparable, suggesting appropriateness of the use of the SN Questionnaire in Japanese participants. No sex difference was observed in the questionnaire (*P* = .588; Supplementary Table S2).

### Other Questionnaire Surveys

The results of the AQ, DASS-21, and REI-40 are summarized in Supplementary Table S2. The score on the AQ was 20.6 ± 1.14 in the current study group. There were no differences between male and female subjects in these scores (*P* = .833; Supplementary Table S2). The stress, anxiety, and depression scores assessed with DASS-21 questionnaire were 7.48 ± 0.82, 3.95 ± 0.54, and 6.91 ± 0.84, respectively, and no sex differences were observed in these scores (*P* = .125, .630, and .056 for stress, anxiety, and depression, respectively; Supplementary Table S2). In the REI-40 for assessments of analytical versus intuitive thinking styles, significant differences between male and female subjects were observed selectively in the rationality subscales (Supplementary Table S2).

### Sucrose–Color Preference Test

In the SCP test, almost all participants answered that the sweetness levels of the 8 sucrose solutions were different and were able to select 1 solution as the sweetest and another as the least sweet. Although the differences did not reach statistical significance, participants overall tended to choose sucrose solutions with colors that were closer to their favorite colors as the sweetest (SCP_m_) and sucrose solutions with colors that were more distant from their favorite colors as the least sweet (SCP_l_; Z = 1.78; *P* = .076; Supplementary Figure S1B and C), suggesting biases in sensory perception. No sex difference was observed in either SCP_m_ (*P* = .620) or SCP_l_ (*P* = .983; Supplementary Table S3).

### Yoni Test

In the Yoni test, participants performed better in cognitive than affective ToM trials (χ ^2^ [N = 38, df = 2] = 24.5 [*P* < .001]; Z = 4.50; *P* < .001 for cYoni vs. aYoni), although reaction time was not different between the trials (χ ^2^ [N = 38, df = 2] = 52.0 [*P* < .001]; Z = 1.72; *P* = .086 for cYoniRT vs. aYoniRT; Supplementary Figure S2A and C; Supplementary Table S3). The percentages of correct responses also significantly declined, and reaction time became longer in the second-order ToM trials compared to those in the first-order trials (Z = 4.46 [*P* < .001] for cYoni first- vs. second-order trials; Z = 4.76 [*P* < .001] for aYoni first- vs. second-order trials; Z = 4.91 [*P* < .001] for cYoniRT first- vs. second-order trials; Z = 5.46 [*P* < .001] for aYoniRT first- vs. second-order trials; Supplementary Figure 2B and D; Supplementary Table S3). No sex difference was observed in either the percentage of correct responses or the reaction time for the first- and second-order cognitive and affective ToM trials of the test (Supplementary Table S3).

### Multiple Regression Analysis for the SB Test and SN Questionnaire

Correlations were substantially high between the measurements within the same questionnaires (e.g., between DASS_t_ and other DASS subscales; between ability and engagement subscales in REI-40) or in the psychological test (e.g., between percentages of correct responses and reaction times in the Yoni test), but not across different questionnaires or psychological tests (Figure 2A). Considering such correlations, SC, NSC, and SN scores were selected for the dependent variables, and associations were analyzed with the following independent variables: AQ, DASS_s_, DASS_a_, DASS_d_, REI_r_, REI_e_, SCP_m_, SCP_l_, cYoni, and aYoni. The backward stepwise analysis found statistically significant models for SC, NSC, and SN. In the model for SC, AQ, DASS_a_, REI_r_, cYoni, and aYoni were identified as substantial contributors, whereas AQ, DASS_a_, DASS_d_, SCP_m_, SCP_l_, and cYoni were associated with NSC (Table 1; Figure 2B). In the model for SN, DASS_a_, DASS_d_, REI_r_, REI_e,_ and cYoni were associated with SN (Table 2; Figure 2B).

**Table 1.** Multiple Regression Analysis for the SB Test

	Unstandardized coefficients		Standardized coefficients			Collinearity	
	B	SEM	Beta	T	Significance	Tolerance	VIF
Model for SC	R = 0.654, R^2^ = 0.427, adjR^2^ = 0.335, F_5,31_ = 4.63, *P* = .003*						
	Residual analysis:						
	Shapiro-Wilk W = 0.981, *P* = .768; Durbin-Watson d = 1.90; Cook’s distance < 0.199; Levene F_1,74_ = 0.253, *P* = .616						
AQ	0.005	0.002	0.379	2.387	0.024*	0.727	1.38
DASS_a_	0.007	0.004	0.279	1.84	0.076	0.801	1.25
REI_r_	0.106	0.026	0.635	4.04	<0.001*	0.748	1.34
cYoni	−0.918	0.357	−0.463	−2.57	0.015*	0.567	1.76
aYoni	0.629	0.282	0.390	2.23	0.033*	0.604	1.66
Model for NSC	R = 0.635, R^2^ = 0.403, adjR^2^ = 0.284, F_6,30_ = 3.38, *P* = .011*						
	Residual analysis:						
	Shapiro-Wilk W = 0.979, *P* = .702; Durbin-Watson d = 2.15; Cook’s distance < 0.137; Levene F_1,72_ = 0.520, *P* = .473						
AQ	0.006	0.003	0.326	2.00	0.055	0.748	1.34
DASS_a_	0.011	0.007	0.284	1.52	0.139	0.572	1.75
DASS_d_	−0.006	0.005	−0.220	−1.13	0.269	0.519	1.93
SCP_m_	0.003	0.001	0.358	2.36	0.025*	0.866	1.15
SCP_l_	0.003	0.001	0.417	2.73	0.001*	0.852	1.17
cYoni	−1.512	0.462	−0.501	−3.28	0.003*	0.850	1.18

Models used the backward stepwise deletion of factors with F = 1.0 to remove and F = 11.0 to enter, respectively. Abbreviations: AQ, Autism-Spectrum Quotient; aYoni, affective assessment of the Theory of Mind; cYoni, cognitive assessment of the Theory of Mind; DASS_a_, Depression Anxiety Stress Scale anxiety subscale; DASS_d_, Depression Anxiety Stress Scale depression subscale; NSC, nonsocial judgement condition; REI_r_, Rational-Experiential Inventory rationality scale; SB, Social Bias test; SC, social condition; SCP_l_, Sucrose–Color Preference least sweet response; SCP_m_, Sucrose–Color Preference most sweet response; SEM, standard error of the mean; VIF, variance inflation factor.

*Statistically significant.

**Table 2.** Multiple Regression Analysis for the SN Questionnaire

	Unstandardized coefficients		Standardized coefficients			Collinearity	
	B	SEM	Beta	T	Significance	Tolerance	VIF
Model for SN	R = 0.606, R^2^ = 0.368, adjR^2^ = 0.266, F_5,31_ = 3.60, *P* = .011*						
	Residual analysis:						
	Shapiro-Wilk W = 0.970, *P* = .391; Durbin-Watson d = 1.60; Cook’s distance < 0.181; Levene F_1,74_ = 0.003, *P* = .953						
DASS_a_	−0.165	0.077	−0.410	−2.14	0.041*	0.554	1.80
DASS_d_	0.157	0.054	0.589	2.09	0.007*	0.494	2.02
REI_r_	0.841	0.453	0.327	1.86	0.073	0.659	1.52
REI_e_	0.783	0.361	0.334	2.17	0.038*	0.862	1.16
cYoni	6.897	4.439	0.225	1.55	0.130	0.969	1.03

Models used the backward stepwise deletion of factors with F = 4.0 to remove and F = 11.0 to enter, respectively. Abbreviations: cYoni, cognitive assessment of the Theory of Mind; DASS_a_, Depression Anxiety Stress Scale anxiety subscale; DASS_d_, Depression Anxiety Stress Scale depression subscale; REI_e_, Rational-Experiential Inventory experientiality scale; REI_r_, Rational-Experiential Inventory rationality scale; SEM, standard error of the mean; SN, Social Norm; VIF, variance inflation factor.

*Statistically significant.

These results suggest that cognitive ToM and anxiety may be comprehensively involved in all social and nonsocial judgments and social conformity, whereas other factors, such as autistic traits, depressive mood, and decision-making styles, may be more selectively involved in these social schemas.

### Path Analysis with Structural Equation Modeling

To further investigate the relationships of the measurements, a path analysis was conducted. First, we considered several models with the layered structures, in which some factors in the first layer, such as autistic traits (AQ) and negative affects (DASS), affected psychological processes, such as ToM and decision making (REI), which in turn affected SC, NSC, and SN. Although the path could still be converged in these layered models, none of them reached the criteria, and the null hypothesis of “the layered models we predicted and the observed data were equal” was rejected (Supplementary Figure S3). We then considered another structure of the model without layers, so that all measurements directly contributed to SN, NSC, and SN (Figure 2C). The nonlayered model successfully fulfilled the criteria. In this model, path coefficients between SC, NSC, and SN were small and insignificant (Figure 2C).

These results suggest that the measurements, such as autistic traits, negative affects, decision-making styles, and ToM, may independently contribute to the social and nonsocial judgments and social conformity, although contributions of the measurements could partly be overlapping.

## Discussion

In this study, we investigated the psychological factors associated with social schemas producing social biases. To do so, a battery of questionnaires and psychological tests were administered in healthy adult subjects, and a multiple regression analysis and structural equation modeling were conducted with these measurements. We found that several factors, such as cognitive ToM and anxiety, were mutually but independently involved in social judgments and conformity, along with several cognitive and affective factors selectively associated with them. Moreover, social judgments are a different process from that of social conformity.

There are several major limitations in the current study. The most important and clearest limitation is the sample size. In this study, only 42 participants were recruited, which could be relatively small, especially for a multiple regression analysis and structural equation modeling, in which a larger sample size is crucial for reliable results. Another limitation is an arbitrary nature of selection of the measurements associated with the social schemas. We selected AQ, analytical versus intuitive decision-making styles, negative affects, and ToM, based on past literature. However, other psychological factors could indeed play imperative roles. In addition, a cultural difference of social conformity has been demonstrated (Bond and Smith, 1996), which is suggested to partly be associated with a difference in the allele prevalence of serotonin transporter genes between regions (Chiao and Blizinsky, 2010). Thus, the findings in Japanese participants in this study may be different from those conducted in people with Western cultural backgrounds. Future studies for replication of the current findings with larger sample sizes and participants with different cultural backgrounds, along with further elucidation of what cognitive and affective mechanisms are involved in social judgments and conformity, are needed.

Studies have shown that negative affects, such as stress and anxiety, are associated with cognitive biases, with higher negative affects heightening biases (Bodenhausen et al., 1994; Harding et al., 2004; Engelmann and Hare, 2018). Accordingly, anxiety was found to be associated with all social and nonsocial judgments and social conformity in this study. The associations of anxiety with social and nonsocial judgments were positive; however, strikingly, the association was negative with social conformity, which was opposite to what was expected based on the previous studies. Similarly, REI_r_, which indicates an analytical decision-making style, was also positively associated with social judgements, and this is also opposite to what was expected, especially given that cognitive and affective ToM were negatively and positively associated with social judgments, respectively, suggesting that ineffective analytical and stronger intuitive processes result in stronger biases in social judgments. Such inverse associations from the expectations are suggested to be sometimes produced by violations of the assumptions for a regression analysis (O’Brien, 2007; Oke et al., 2019). Nonetheless, we utilized reasonably stricter criteria for collinearity than what had been recommended (O’Brien, 2007; Oke et al., 2019). Moreover, outliers were well controlled, and the assumptions of multivariate normality, autocorrelation, and homoscedasticity were also met with the criteria (Harrell and Lee, 1985; Kramer, 1985). Thus, the inverse associations from the predictions observed in this study may require other explanations, such as opposite directions of the relationships. For instance, although anxiety may heighten social conformity, conforming to social norms could alleviate anxiety.

Previous studies by Lavin and Lawless (Lavin and Lawless, 1998) and Bayarri and colleagues (2001) have demonstrated that colors affect the perception of sweetness. Taking advantage of this, we conceived the SCP test to assess cognitive bias on sensory perception. A novel finding in this test was that participants tended to rate sucrose solutions with the colors they preferred as sweeter than those with nonpreferred colors, although this difference did not reach statistical significance, since a substantial number of participants rated contrariwise. However, it is important to note that almost all participants still rated the sweetness of sucrose solutions as different across different colors, although the concentration of sucrose solutions was identical, and participants were instructed to answer that sweetness was equal for all solutions if they desired. In particular, associations were observed between the measurements of the SCP test (SCP_m_ and SCP_l_) and nonsocial judgments (NSC), but not social judgments (SC) and social conformity, which is consistent with the theory that nonsocial judgments may be associated with nonsocial cognitive bias, and this process could be distinct from social judgments and conformity.

Accumulating evidence suggests the existence of ToM deficits (Baron-Cohen et al., 1985; Frith, 2012) and altered cognitive bias (Brosnan et al., 2016) in ASD. In the view of analytical versus intuitive processes in the dual process theory, ASD subjects appear to be more analytical- and less intuitive-oriented than normal subjects, as ASD subjects exhibit impairments in tests that require intuitive reading of the emotional expressions of others, such as those determining another’s thoughts based on their eye gaze (Ogawa et al., 2017), whereas ASD subjects have higher mathematical aptitudes than normal subjects (Iuculano et al., 2014). Although cognitive and affective ToM have not yet been closely tested in ASD subjects, since ASD subjects exhibit impairments in both reading emotional facial expressions (Wang et al., 2007; Uljarevic and Hamilton, 2013; Ogawa et al., 2017) and attributing beliefs and thoughts to others (Baron-Cohen et al., 1985; Frith, 2012), it is likely that ASD encompasses both cognitive ToM and affective ToM deficits. However, this is not entirely consistent with the view of ASD from the dual process theory indicating an analytical and intuitive imbalance. In this study, we administered the AQ questionnaire, attempting to gain some insight in relation to this issue. We found that social judgments were associated with both cognitive and affective ToM, with cognitive ToM negatively associated and affective ToM positively associated, suggesting that poor attribution of thoughts and stronger recognition of the emotional states of others cause more biases on social judgements. AQ results were associated with both social and nonsocial judgments but not social conformity, suggesting that autistic traits may be more robustly involved in judgements of others, but less in decision-making of one’s own social behaviors.

Alterations of nonsocial cognitive biases have been reported in some psychiatric disorders, such as ASD (Brosnan et al., 2016; Brosnan et al., 2017) and depression (Murrough et al., 2011), whereas only a few, if any, studies have examined whether social biases may be altered in psychiatric disorders and, if altered, how they may be associated with their symptoms. In this study, we found that ToM and negative affects, which are psychological processes impaired by or involved in psychiatric disorders, such as ASD, schizophrenia, and depression (Brune and Brune-Cohrs, 2006; Ang and Pridmore, 2009; Derntl et al., 2012; Hagele et al., 2016), were associated with social schemas pertinent to social biases, suggesting the possibility of altered social biases in these psychiatric disorders. Further investigations unveiling social biases in subjects with these psychiatric disorders promise to yield some novel insights in our understanding, and thereby treatments, of the disorders.

The neural mechanisms underlying social biases have also remained unclear, and further investigations are imperative. The current study found that cognitive ToM was associated with all social and nonsocial judgements and social conformity. Social conformity was also associated with negative affects, whereas social judgement was associated with affective ToM and AQ. Neuroimaging studies have shown that both cognitive and affective ToM involve activation of the classical ToM network, consisting of brain regions such as the posterior superior temporal sulcus, precuneus, temporal poles, and supplementary motor area (Sebastian et al., 2012; Bodden et al., 2013), suggesting that the classical ToM network may underlie processing of social and nonsocial judgements, as well as social conformity. The studies have further demonstrated additional activation of the orbitofrontal cortex (OFC; Bodden et al., 2013) and ventromedial prefrontal cortex (vmPFC; Sebastian et al., 2012) in affective ToM. A lesion in the vmPFC and OFC has also been reported to impair affective ToM in human subjects (Shamay-Tsoory and Aharon-Peretz, 2007; Shamay-Tsoory et al., 2010). Moreover, the AQ score is also correlated with a reduction of cortical thickness in the OFC (Gebauer et al., 2015). These studies suggest that interactions between the classical ToM network and the OFC/vmPFC may be involved in social judgments. The association of social conformity with negative affects suggests that social conformity may involve limbic structures such as the amygdala, whose activation is associated with all anxiety, depression, and stress (Anand and Shekhar, 2003; Rauch et al., 2003; Zhang et al., 2021); thereby, interactions between the classical ToM network and amygdala may play a role in social conformity.

In conclusion, our study suggests that negative affects and ToM may be involved in social biases, which may, be roughly distinguished into 2 types: 1 accompanying social judgments, which is more strongly associated with cognitive processes, and the other accompanying social conformity, which is more strongly associated with affective processes.

## Supplementary Material

pyab022_suppl_Supplementary_Material
