# Supplementary material for: Cognitive and Affective Processes Associated with Social Biases
Source: Int J Neuropsychopharmacol. 2021 Apr 30;24(8):645–55. doi: 10.1093/ijnp/pyab022 (PMC8378077; doi:10.1093/ijnp/pyab022)
Supplement: pyab022_suppl_Supplementary_Material [file pyab022_suppl_supplementary_material.pdf]

Manuscript Title:
Cognitive and Affective Processes Associated with Social Biases

Authors:
A. Kaneko, Y. Asaoka, Y-A. Lee & Y. Goto

Supplementary Table S1
Sample structure

Supplementary Table S2
A summary of measurements with questionnaires

Supplementary Table S3
A summary of measurements for Sucrose-Color Preference and Yoni tests

Supplementary Figure S1
Sucrose-Color Preference (SCP) test

Supplementary Figure S2
Yoni test

Supplementary Figure S3
Path analysis with the layered structure model

Supplementary Table S1. Sample structure

Gender	N	Age				eFIQ			
		Min	Max	Mean	s.e.m.	Min	Max	Mean	s.e.m.
**Male**	20	20	29	23.6	0.45	88	130	105.4	2.53
**Female**	22	18	47	25.0	1.56	94	127	105.6	1.89
Σ	42	18	47	24.3	0.84	88	130	105.5	1.54

eFIQ - Estimated full scale intelligence quotient

Supplementary Table S2. A summary of measurements with questionnaires

	Index	Σ (n=42)	Normality		Male (n=20)	Female (n=22)	Male vs. Female	
			Shapiro-Wilk W	p			t or Z[#]	p
**Social Norm Questionnaire**								
Total score	SN	17.6 ± 0.21	0.938	0.025	17.8 ± 0.31	17.5 ± 0.29	0.542	0.588
**Autism Quotient**								
Total score	AQ	20.6 ± 1.14	0.967	0.267	20.9 ± 1.74	20.4 ± 1.53	0.212	0.833
**DASS-21 Questionnaire**								
Total score	$DASS_t$	18.3 ± 1.91	0.945	0.044	15.4 ± 2.39	21.0 ± 2.85	−1.399	0.162
Stress	$DASS_s$	7.48 ± 0.82	0.974	0.509	6.15 ± 1.10	8.68 ± 1.17	−1.569	0.125
Anxiety	$DASS_a$	3.95 ± 0.54	0.892	0.001	3.25 ± 0.72	4.59 ± 0.78	−0.482	0.630
Depression	$DASS_d$	6.91 ± 0.84	0.909	0.003	6.10 ± 1.15	7.64 ± 1.21	−1.907	0.056
**REI-40 Questionnaire**								
Rationality	REI_r	3.78 ± 0.08	0.975	0.495	4.07 ± 0.09	3.52 ± 0.10	3.854	<0.001*
Experientiality	REI_e	3.12 ± 0.09	0.933	0.017	3.07 ± 0.15	3.16 ± 0.12	−0.505	0.614
Rational ability	$REI_{r\text{-}a}$	3.58 ± 0.09	0.975	0.488	3.71 ± 0.13	3.46 ± 0.12	4.372	<0.001*
Rational engagement	$REI_{r\text{-}e}$	3.98 ± 0.09	0.976	0.527	4.20 ± 0.11	3.79 ± 0.12	2.669	0.011*
Experiential ability	$REI_{e\text{-}a}$	3.05 ± 0.11	0.975	0.479	3.17 ± 0.17	2.95 ± 0.13	−0.021	0.930
Experiential engagement	$REI_{e\text{-}e}$	3.18 ± 0.10	0.927	0.011	3.27 ± 0.17	3.11 ± 0.12	−1.251	0.211

Data expressed as mean ± s.e.m.

*: $p<0.05$ compared to male.

[#]: Unaired t test or Mann-Whiteny U test for the data with $p<0.05$ or $p>0.05$, respectively, in Shapiro-Wilk test.

Supplementary Table S3. A summary of measurements for Sucrose-Color Preference and Yoni tests

	Index	Σ	Normality		Male	Female	Male vs. Female	
			Shapiro-Wilk W	p			t or Z#	p
**Sucrose-Color Preference (SCP) Test** (Sample Number: Σ=41, Male=20, Female=21; 1 outlier removed†)								
Sweetest color distance	SCP_m	24.0 ± 2.39	0.945	0.047	23.6 ± 3.67	24.4 ± 3.17	−0.496	0.620
Least sweet color distance	SCP_l	32.1 ± 2.77	0.962	0.182	32.0 ± 4.05	32.1 ± 3.89	−0.021	0.983
**Yoni Test** (Sample Number: Σ=38, Male=20, Female=18; 1 subject not completed the test; 3 outliers removed†)								
Percentage correct (%)								
Cognitive ToM	cYoni	95.8 ± 0.72	0.833	<0.001	95.8 ± 1.22	95.8 ± 0.75	0.918	0.359
Affective ToM	aYoni	91.3 ± 0.81	0.899	0.002	91.5 ± 1.43	91.1 ± 1.01	0.563	0.574
Physical ToM	pYoni	96.8 ± 0.84	0.655	<0.001	97.1 ± 0.96	96.4 ± 1.44	0.053	0.958
1st order cognitive ToM	$cYoni_1$	100.0 ± 0.00	--	--	100.0 ± 0.00	100.0 ± 0.00	--	--
2nd order cognitive ToM	$cYoni_2$	93.8 ± 1.09	0.833	<0.001	93.8 ± 1.83	93.8 ± 1.13	0.830	0.406
1st order affective ToM	$aYoni_1$	97.4 ± 1.73	0.195	<0.001	97.5 ± 2.10	100.0 ± 0.00	−1.360	0.174
2nd order affective ToM	$aYoni_2$	88.8 ± 1.15	0.882	<0.001	89.4 ± 1.83	88.1 ± 1.34	1.041	0.298
1st order physical ToM	$pYoni_1$	97.0 ± 1.10	0.493	<0.001	96.9 ± 1.54	97.2 ± 1.62	−0.238	0.812
2nd order physical ToM	$pYoni_2$	96.5 ± 1.12	0.502	<0.001	97.5 ± 1.37	95.4 ± 1.81	0.952	0.341
Reaction Time (ms)								
Cognitive ToM	cYoniRT	4735 ± 259.5	0.961	0.200	4889 ± 379.0	4564 ± 357.3	0.622	0.538
Affective ToM	aYoniRT	4538 ± 252.1	0.930	0.020	4584 ± 380.3	4487 ± 335.0	−0.058	0.953
Physical ToM	pYoniRT	2802 ± 94.31	0.977	0.614	2850 ± 157.7	2750 ± 98.39	0.525	0.603
1st order cognitive ToM	$cYoniRT_1$	2841± 238.0	0.728	<0.001	3291 ± 418.5	2341 ± 119.6	1.754	0.079
2nd order cognitive ToM	$cYoniRT_2$	5747 ± 338.6	0.955	0.124	5741 ± 454.3	5755 ± 520.1	−0.020	0.984
1st order affective ToM	$aYoniRT_1$	2887 ± 174.6	0.873	<0.001	3034 ± 292.0	2724 ± 176.6	0.555	0.579
2nd order affective ToM	$aYoniRT_2$	5144 ± 292.1	0.935	0.030	5130 ± 423.7	5160 ± 411.4	−0.292	0.770
1st order physical ToM	$pYoniRT_1$	2207 ± 106.3	0.965	0.282	2291 ± 175.4	2114 ± 112.7	0.827	0.414
2nd order physical ToM	$pYoniRT_2$	3599 ± 135.0	0.979	0.685	3590 ± 233.0	3608 ± 127.6	−0.065	0.949

Data expressed as mean ± s.e.m.

#: Unaired t-test or Mann-Whiteny U test for the data with p<0.05 or P>0.05, respectively, in Shapiro-Wilk test.

†: Outliers removed by the criteria of x_i< or >Mean±3SD for the data with normal distribution, and x_i< or >M±3MAD for the data with non-normal distribution.

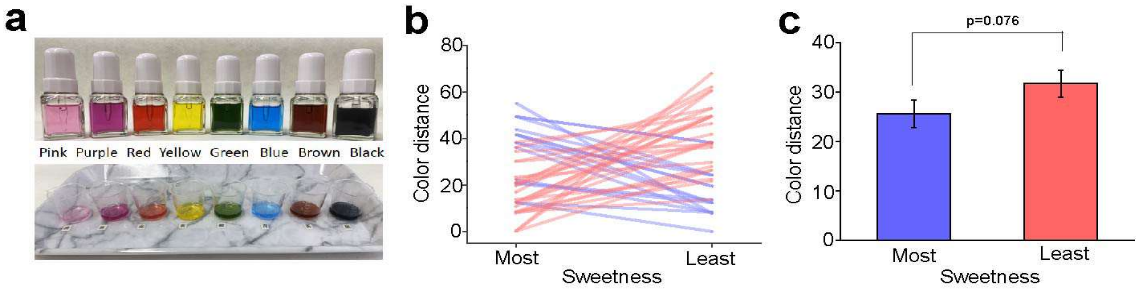

Supplementary Figure S1. Sucrose-Color Preference (SCP) test. **(a)** Photos illustrating colored sucrose solutions used in the test. **(b)** A graph showing the color distance between the color that they preferred and the color they rated the sucrose solution the sweetest (Most) and least sweet (Least), respectively, in individuals. Red and blue lines indicate subjects whose color distance for the Least is larger than the Most and vice versa, respectively. **(c)** A bar graph showing the color distance but averaged for all subjects.

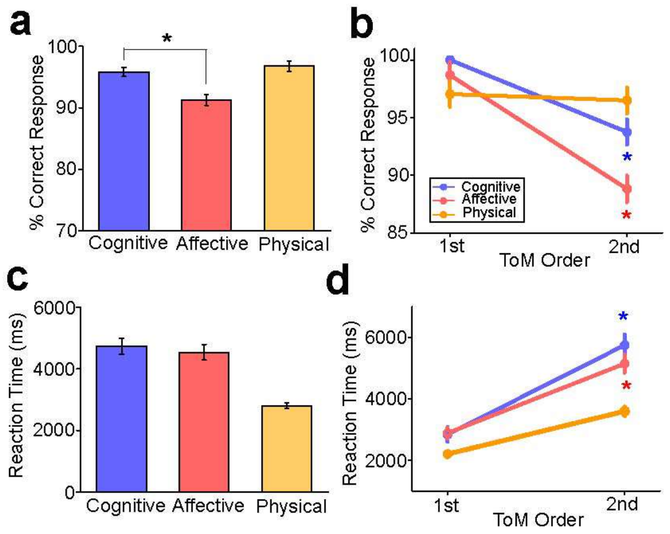

Supplementary Figure S2. Yoni test. (a) A graph showing percentage correct responses for each of cognitive, affective, and physical ToM trials in the test. *p<0.001, Friedman ANOVA with post-hoc Wilcoxon Rank Sum test. **(b)** A graph showing percentage correct responses for each of the 1st and 2nd order ToM of cognitive, affective, and physical trials in the test. *p<0.001, compared to 1st order. **(c, d)** Graphs similar to (a) and (b) but showing reaction time in the test.

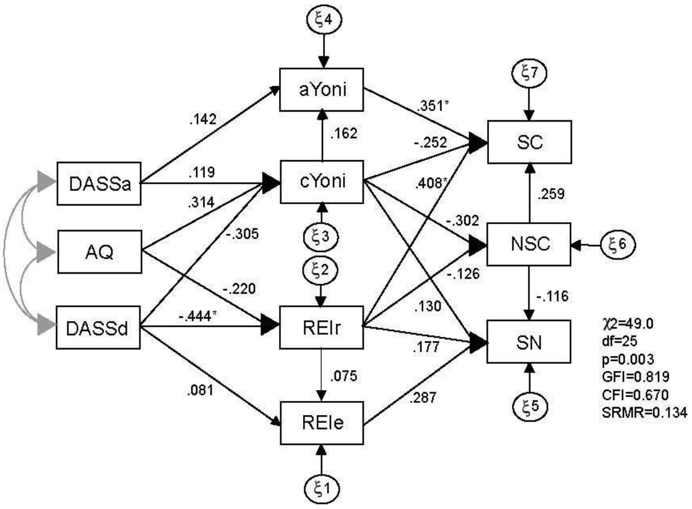

Supplementary Figure S3. Path analysis with the layered structure model. A Path diagram illustrating the layered structure model with the best fitting values of chi-square statistic, GFI, CFI, and SRMR, but still has not been met with the criteria for a just identified or saturated model. *p<0.05 for path coefficients. Index: AQ, Autism-spectrum Quotient score; $DASS_a$, Anxiety score in Depression Anxiety and Stress Scale; $DASS_d$, Depression score in Depression Anxiety and Stress Scale; cYoni, Percentage corrects in cognitive ToM of Yoni test; aYoni, Percentage corrects in affective ToM of Yoni test; REI_r, Rationality score in Rational-Experiential Inventory; REI_e, Experientiality score in Rational-Experiential Inventory; SC, Discrimination index score for social judgements in the Social Bias test; NSC, Discrimination index score for non-social judgements in the SB test; SN, Total score in the Social Norm Questionnaire, GFI, Goodness-of-fit index; CFI, Comparative fit index; SRMR, Standardised root mean square residual.
